# Association of Access to Healthcare with Self-Assessed Health and Quality of Life among Old Adults with Chronic Disease in China: Urban Versus Rural Populations

**DOI:** 10.3390/ijerph16142592

**Published:** 2019-07-20

**Authors:** Tao Zhang, Chaojie Liu, Ziling Ni

**Affiliations:** 1 Department of Health Management, School of Medicine and Health Management, Tongji Medical College, Huazhong University of Science and Technology, Wuhan 43003, China; 2 School of Psychology and Public Health, La Trobe University, Melbourne 3086, Australia

**Keywords:** Self-assessed health, Quality of life, Access to healthcare, The elderly, Chronic diseases, Rural, China

## Abstract

This study examined urban–rural differences in the association of access to healthcare with self-assessed health and quality of life (QOL) among old adults with chronic diseases (CDs) in China. The data of 5796 older adults (≥60) with self-reported CDs were collected from the Study on Global Ageing and Adult Health in China, including indicators of self-assessed health and QOL and information on access to healthcare. Associations of access to healthcare with self-assessed health and QOL at the 10th, 50th, and 90th conditional quantiles were determined after controlling individual and household factors, showing that urban patients who received healthcare within two weeks gave higher ratings on self-assessed health scores at the 10th and 50th quantiles. In rural areas, one-year and two-week access to healthcare was found to be associated with QOL scores at the 10th and 90th quantiles, respectively. Marginal effects of using needed health service decreased with a growth in QOL and self-assessed health scores in both urban and rural locations despite these effects being significant across the whole distribution. Overall, access to healthcare affects the self-assessed health and QOL of the elderly with CDs in China, especially in patients with poor health, though differently for urban and rural patients. Policy actions targeted at vulnerable and rural populations should give priority to reducing barriers to seeking health services.

## 1. Introduction

With the transformation of disease, chronic diseases (CDs) have been recognized as the main diseases endangering human health. Of approximately 56 million deaths globally, 60% are due to major chronic disease [1]. This situation is becoming more severe in China due to an aging population. In 2016, there were 230 million elderly people over the age of 60, accounting for 16.7% of the total population, but the proportion of elderly people with CDs was over 65% [2]. It is predicted that the number of the elderly will increase to 450 million by 2050, representing nearly one-third of the total population [3]. Therefore, the Chinese government will face great challenges in the prevention and treatment of CDs in the coming decades. 

CDs are diseases of long duration and generally slow progression that cause 86% of the deaths and 70% of the disease burden in China [4,5]. Compared with other age groups, the elderly (individuals ≥65 years of age) endure a higher risk of suffering from CDs and higher mortality and heavier economic burden of disease [6]. Additionally, previous studies revealed that individuals with CDs have a lower general health status and a lower level of quality of life (QOL) than the general population [1,7,8,9]. Therefore, adequate access to healthcare is especially critical for older adults with CDs who require greater levels of treatment and care to prolong survival and further improve perception of their lives [10]. 

Access to healthcare is defined as timely use of personal health services when needed. In the past few decades, the Chinese government has made efforts to reduce barriers of access to healthcare and provide affordable health services for vulnerable populations, for example by establishing a universal medical insurance system and a catastrophic disease insurance system [11,12]. Moreover, health management for patients with CDs was regarded as a basic service in the National Essential Public Health Services Package [13]. These measures improved the accessibility of healthcare demonstrated in previous studies, especially for seniors [14,15]. In addition, some studies from Western countries have shown a positive association between access to healthcare and health outcomes. For instance, Alonso and his colleagues found that lack of access to adequate health service increased five-year mortality by 80% among those with two or more chronic conditions, and increased mortality by 155% among those who were disabled [16]. Also, prior research indicated that access to healthcare made the greatest difference in slowing functional decline and reducing the risk of death among those elders with no functional limitations at baseline [17].

However, most earlier studies examined the associations of access to healthcare with life expectancy and mortality rate [18] but rarely investigated perceived health or QOL comprehensively and holistically. Moreover, urban and rural residents in China show extreme differences in socioeconomic status and health services utilization [19]. For example, old people residing in a rural location often cannot afford health expenses and are less likely to use inpatient services [20]. Therefore, it is largely unknown whether the associations between access to healthcare and health outcomes in rural and urban areas are different. Additionally, a previous study indicated that people with different levels of health presented varied needs for healthcare [21]. It is therefore assumed that the importance of access to healthcare might differ depending on the level of the health outcome distributions.

This study sought to determine the associations of access to healthcare with self-assessed health and QOL among the elderly with CDs residing in urban and rural areas using national data from China to provide empirical evidence for measures to address issues of accessibility of healthcare.

## 2. Methods

### Data

The data were extracted from the World Health Organization (WHO)-sponsored Study on Global Ageing and Adult Health (WHO-SAGE) conducted from 2007 to 2010 in China. Study participants were selected through a multistage stratified random sampling strategy. The first stage involved a selection of municipalities in eight provinces (Guangdong, Shandong, Zhejiang, Shanghai, Shaanxi, Yunnan, Hubei, and Jilin), and the primary sampling units were then narrowed down to streets/towns using probability sampling proportional to population size. Finally, participating households were selected randomly and those 50 years or older were invited to complete a questionnaire survey. In this survey, a total of 15,050 adults were identified for the individual questionnaires and 14,813 individual interviews were completed, for a 98.4% response rate. Details of the WHO-SAGE study protocol are published elsewhere [22]. 

After data cleaning, 6493 respondents aged 60 or older who reported one or more chronic conditions among the list of arthritis, stroke, heart disease, diabetes, chronic lung disease, asthma, depression, hypertension, cataract, and oral disease remained. Exclusion of the questionnaires containing missing values resulted in a final sample size of 5796 (3194 urban and 2602 rural) for data analysis. 

## 3. Measurements

### 3.1. Outcome Variables

In this study, self-assessed health and QOL served as the outcome variables. For self-assessed health, the WHO-SAGE survey asked respondents to rate their experienced problems in relation to mobility, self-care, pain and discomfort, cognition, interpersonal activities, sleep or energy, affect, and vision in the preceding 30 days on a five-point Likert scale (1 = *none*, 2 = *mild*, 3 = *moderate*, 4 = *severe*, 5 = *extreme*) [23]. The Chinese version of this instrument has been tested in terms of reliability and validity, and more details can be found elsewhere [24].

The QOL was assessed using the World Health Organization Quality of Life (WHOQOL) score. This scale covers eight aspects of life rated on a five-point Likert scale [25], including possession (1 = *completely*, 2 = *mostly*, 3 = *moderately*, 4 = *a little*, 5 = *none at all*) of energy for daily life and money to meet living needs; and satisfaction (1 = *very satisfied*, 2 = *satisfied*, 3 = *neither satisfied nor dissatisfied*, 4 = *dissatisfied*, 5 = *very dissatisfied*) with health, self, ability to perform daily activities, personal relationships, condition of living space, and overall QOL. This scale has an acceptable reliability and validity and has been used widely. Details of how this scale was developed, validated, and adapted for use in this survey are described elsewhere [24]. 

Coding for these items was reversed and a summed score was calculated for self-assessed health and QOL. The summed scores were then transformed to a score ranging from 0 to 100, with a higher score indicating better health and QOL (Table 1).

### 3.2. Explanatory Variables

Access to healthcare was the major interest of this study. Use of health services can be shaped by a wide range of factors, including the availability, accessibility, affordability, accommodation, and acceptability of services [26,27]. Health service surveys usually asked respondents to report their use of outpatient services over the past weeks and inpatient services over the past year [17,28]. Such an approach is unlikely to capture those who have access to healthcare when in need, but not within the window of time covered by the surveys. 

In this study, we examined the access to healthcare of those with chronic conditions. Theoretically, all patients with CDs would need certain care, either for preventive or for therapeutic and rehabilitative purposes. In the WHO-SAGE, respondents were asked (1 = *yes*, 0 = *no*): (1) Have you been taking any medications or other treatment in the last two weeks? (2) Have you been taking any medications or other treatment in the last 12 months? (3) The last time you needed health care, did you get the care?

### 3.3. Control Variables

Health and QOL can be determined by many factors [23,27,29]. The selection of control variables in this study considered the Grossman health demand model [30], in which individual, family, and social activities are considered as inputs in the health production function; and the Andersen health behavioral model [29], where socioeconomic variables are deemed as enabling factors for health behaviors and use of health services.

The WHO-SAGE collected data on gender, age, marital status, ethnicity, educational attainment, and living and working conditions of each individual respondent and their health risk behaviors such as smoking (current), alcohol consumption (over the past 30 days), and physical activities (over the past week). Physical activities were measured by weekly engagement in vigorous or moderate- intensity sports, fitness, or recreational (leisure) activities that cause an increase in breathing or heart rate for at least 10 minutes at a time.

Financial status and social support were measured at the household level, including household size, regular source of income, debts, and healthcare expenditure as a percentage of household income. Respondents were also asked to rate their overall household finances on a five-point Likert scale (*very good, good, moderate, bad, very bad*) and report whether they received any financial or in-kind support from the government or from clubs or groups in the community (Table 1). 

### 3.4. Statistical Analysis

The characteristics of respondents were described using mean ± SD (standard deviation) for continuous variables and number and percentages (%) for categorical variables. The differences between urban and rural participants in these variables were tested using a *t*-test and chi-square test. Quantile regression models were established to determine the associations of access to healthcare with self-assessed health and QOL after adjustment for variations in the control variables [31]. Unlike the ordinary least square (OLS) model that depends only on conditional means, regression models on conditional quantile functions are particularly useful for variables with heterogeneous conditional distributions [32,33].

The specific quantile of the outcome distribution conditioned by the values of the predictor variables is given by:

$$Q_{yi}\left[\tau |x_{1i},x_{2i},\ldots ,x_{ki}\right]=\beta_{0}\left(\tau \right)+\beta_{1}\left(\tau \right)x_{1i}+\beta_{2}\left(\tau \right)x_{2i}+\cdots +\beta_{k}\left(\tau \right)x_{ki}$$

where $Q_{yi}\left(\tau \;|\right)$ denotes the τ-th (0 < τ < 1) quantile of the conditional distribution of $y_{i}$, the outcome variable (self-assessed health and QOL). The regression parameter $\beta_{k}\left(\tau \right)$ denotes how the specified quantile changes with a one-unit change in $x_{k}$. In order to observe the association between access to healthcare and low-, middle-, and high-level health, we selected three different distributions of self-assessed health and QOL: 10th, 50th, and 90th percentiles. Separate models were established for urban and rural residents. A *p*-value of less than 0.05 was considered statistically significant. Weights to account for the sampling design of the WHO-SAGE were used in analytical models. All analyses were performed using STATA 14.0 (StataCorp LP., College Station, TX, USA).

## 4. Results

### 4.1. Characteristics of Respondents

The urban respondents had mean scores of 89.29 and 71.91 for self-assessed health and QOL, respectively, compared with 86.79 and 69.46 for their rural counterparts. Most respondents (93%), both urban and rural, obtained healthcare services when needed, but only slightly more than half of the respondents had taken medications or received other treatment for their chronic conditions over the past 12 months. This figure decreased further to 39% for the urban and 29% for the rural respondents over the preceding two weeks. 

Overall, the demographic characteristics (gender, age, and marital status) and other aspects of the urban and rural respondents were significantly different. For example, the urban respondents attended school for more years, were more likely to move residence, and had a healthier lifestyle (less smoking and drinking and more vigorous exercise). Although the urban respondents reported better financial situations, they spent more on healthcare as a percentage of household income than their rural counterparts.

### 4.2. Associations of Access to Healthcare with Self-Assessed Health and QOL in Urban Respondents

The regression coefficients of the health access variables varied across the three quantiles (10th, 50th, and 90th) of self-assessed health and QOL, implying that the associations depended on the distribution levels of the self-assessed health and QOL (Table 2).

Access to healthcare for chronic conditions over the past two weeks and recent use of healthcare when needed were positively associated with self-assessed health at the 10th and 50th quantiles. Although recent use of healthcare when needed was positively associated with QOL across all three quintiles, greater regression coefficients were found for the lower quantiles (Table 2). Notably, receiving health services within one year showed no significant effect on self-assessed health and QOL at the three quantiles.

**Figure 1:**
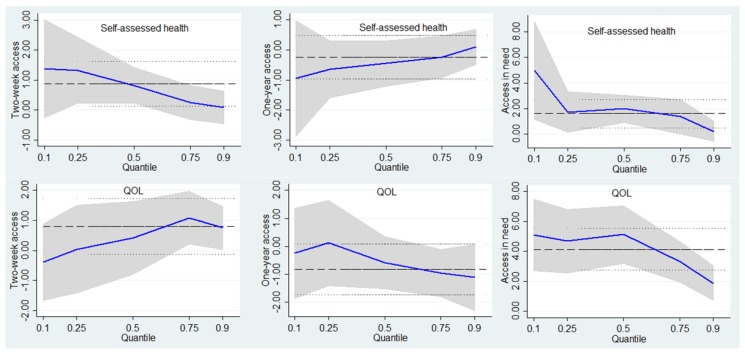
Point estimates and 95% confidence intervals from a quantile regression of the self-assessed health and QOL distribution for urban respondents.

Consistent with the estimation above, Figure 1 shows that two-week access and recent use of healthcare when needed were expected to be more important when self-assessed health and QOL were lower in the urban respondents.

### 4.3. Associations of Access to Healthcare with Self-Assessed Health and QOL in Rural Respondents

Similar to the regression models for urban respondents, recent use of needed healthcare was positively associated with self-assessed health in rural respondents, more so when self-assessed health was lower. However, access to healthcare for chronic conditions over the past two weeks was found to be positively associated with QOL only at the 90th quantile. In contrast, access to healthcare for chronic conditions over the past 12 months was positively associated with QOL only at the 10th quantile (Table 3).

**Figure 2:**
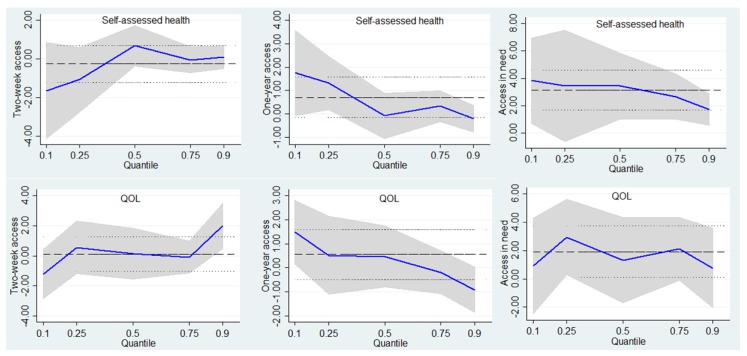
Point estimates and 95% confidence intervals from a quantile regression of the self-assessed health and QOL distribution for rural respondents.

Although access to healthcare in general was positively associated with self-assessed health and quality of life, the roles of the three access indicators appeared to vary between the urban and rural respondents (Figure 2). First, receipt of healthcare within one year had a significant effect on rural but not urban residents with low quality of life. Second, two-week access only explained the variation of health status at the 10th and 50th quantiles in an urban setting, but this indicator seems significant for rural respondents with high health. 

## 5. Discussion

Access to healthcare is regarded as a vital resource to ensure patients receive timely health services and maximize health outcomes. This study focused on the relationships of access to healthcare with self-assessed health and QOL in the urban and rural elderly with CDs using data from a nationwide survey in China. Consistent with other studies [17,18,27], realizing access to care made a substantial difference in self-perceived health and QOL after controlling individual and household level factors, despite these relationships varying between urban and rural areas.

In Chinese urban areas, receiving health care within two weeks was found to be significantly related to patients with low and medium health status. Indeed, most old patients suffering from CDs need long-term and continuous healthcare services [34]. As a result, those who received regular and timely care can recover physiological function, which is helpful to improving their perception of health status. However, this association is not significant among the population with high-level health, who unlike their counterparts with poor health, understandably do not have high needs for health services. Therefore, they are less likely to use health services more frequently, consequently reducing the impact of care [35].

Unlike urban respondents, those from rural regions who received healthcare within 12 months and two weeks demonstrated a positive relationship with QOL at low quantile and high quantile, respectively. Although the healthcare system in rural China has been greatly improved and most rural adults with health problems can receive health services, the great gap between rural and urban populations in access to healthcare still exists [36]. In rural locations, lack of pensions, lower income, higher out-of-pocket costs, and greater co-payments are still regarded as critical financial barriers to adequate health services [19]. Therefore, those who accessed regular sources of healthcare even within one year also tend to express greater satisfaction with their lives than other rural residents. However, providing health services within a short period (such as two weeks) appeared to be more important for those with high QOL, because this part of the population in general has a higher need for healthcare, such as higher frequency of service [35].

Comparison of urban and rural respondents’ responses on access to healthcare suggests that provision of regular health services seems to have a greater effect on old adults residing in rural areas. According to a previous study [18], most rural older adults still regarded financial constraints as the main barrier to medical services. Additionally, elderly people in rural areas also experienced difficulty in seeking healthcare from family members since more young people have migrated to urban areas for a better life under the situation of accelerated urbanization in China [37]. On the other hand, older adults in urban locations generally benefit from convenient health care such as home-bed services due to the fact that large amounts of health resources are distributed here [19].

Consistent with another study [38], the marginal effects of using health services when needed decreased with a growth in QOL and self-assessed health scores in both urban and rural locations, whereas these effects were observed to be significant across the whole distribution. QOL and perceived health reflect a comprehensive experience of one’s physical state, mental function, social ability, and overall personal situation [39,40]. Providing appropriate health services based on individual needs is beneficial to health through diagnosis and treatment at an early stage and by further facilitating improvements in physiological and psychological functions [18,41]. However, these influences are not strong for those with better health status, possibly due to fact that this population is supposed to have higher expectations and stronger requirements on healthcare, such as quality and safety, rather than just receiving healthcare when needed [42].

Notably, receipt of health services within 12 months showed no obvious association with either self-assessed health or QOL across the entire distribution in urban regions. One potential interpretation is that current access to healthcare can only affect the current level of health capital according to a theoretical perspective from health economics [27,43], which therefore suggests that one-year access has a weak influence on the individual current health we measured. In addition, older adults in urban areas of China, unlike rural ones, can receive adequate healthcare. Consequently, the majority of the elderly with CDs can access health services within 12 months, which resulted in the lack of sensitivity of this variable in the model.

With increasing growth of the elderly population and incidence of CDs in China, providing regular and timely healthcare is viewed as a vital measure to improve life expectancy and health expectancy. Several implications for health policy may be considered from this study. First, equalization of essential health services should receive priority to reduce barriers to healthcare, especially for rural residents [44]. Additionally, an affordable basic health service package should be developed for older adults needing long-term care [27], such as regular blood pressure measurements for hypertensive patients and follow-up and intervention services for diabetic patients. Moreover, spatial accessibility should be taken under consideration, particularly in the remote and underdeveloped areas of China [45]. 

Our study has some limitations. First, an evident limitation is that cross-sectional data generally make causal inference problematic. Second, we used a single subjectively rated assessment of self-assessed health and QOL in the WHO-SAGE, which may introduce some bias since individual expectations or conceptualizations might differ. Third, community or regional factors potentially influencing health (such as percentage of free public medical services) were not included in the control variables due to lack of relevant data in WHO-SAGE, despite our concern for the influences of individual and household-level factors on health, which may be the reason for the low pseudo *R*^2^ at the upper quantile. Fourth, caution needs to be taken when generalizing the findings to present circumstances since the data we used are not up to date. 

In spite of these shortcomings, we have extended current research by focusing on the older population with CDs in urban and rural locations using a nationally representative sample. Furthermore, this analysis, unlike other studies, gives more insight into the association of access to healthcare with health outcomes through the use of quantile regression models.

## 6. Conclusions

Generally, access to healthcare is positively correlated with the self-assessed health and QOL of the elderly with CDs in China, especially for people with low-level health. However, the impact of access to healthcare appeared to vary between the urban and rural groups. Under the universal healthcare coverage in China, improving the affordability of medical care and reducing barriers to healthcare should be given priority to afford the elderly securer access to basic healthcare services. 

## Figures and Tables

**Table 1:** Description of variables and general characteristics of respondents.

Variables	Description	Urban (*n* = 3194)	Rural (*n* = 2602)	*p*
Outcome variables				
Self-assessed health	Mean ± SD	89.29 ± 8.96	86.79 ± 10.05	<0.001
Quality of life	Mean ± SD	71.91 ± 11.10	69.46 ± 12.21	<0.001
Explanatory variables				
Two-week access	To medications or treatment for chronic conditions over the past 2 weeks (*n*, %)	1247 (39.00)	750 (28.80)	<0.001
One-year access	To medications or treatment for chronic conditions over the past 12 months (*n*, %)	1751 (54.80)	1318 (50.70)	0.002
Access in need	Received healthcare services when needed (*n*, %)	2970 (93.00)	2410 (92.60)	0.592
Control variables				
Sex	Male (*n*, %)	1356 (42.50)	1179 (45.30)	0.029
	Female (*n*, %)	1838 (57.50)	1423 (54.70)	
Age	Years (mean ± SD)	65.81 ± 9.39	63.76 ± 9.00	<0.001
Marital status	Never married (*n*, %)	22 (0.70)	19 (0.70)	0.029
	Currently married (*n*, %)	2589 (81.10)	2102 (80.80)	
	Cohabiting (*n*, %)	6 (0.20)	7 (0.30)	
	Separated/divorced (*n*, %)	62 (1.90)	25 (1.00)	
	Widowed (*n*, %)	515 (16.10)	449 (17.30)	
Ethnicity	Han (*n*, %)	3131 (98.00)	2577 (99.00)	0.002
	Others (*n*, %)	63 (2.00)	25 (1.00)	
Education	Years of school (mean ± SD)	7.42 ± 4.67	3.09 ± 3.25	<0.001
Living arrangement	Moved from somewhere else (*n*, %)	2271 (71.10)	779 (29.90)	<0.001
	Always lived in the same village/town/city (*n*, %)	923 (28.90)	1823 (70.10)	
Work	At least two days during the last seven days (*n*, %)	2590 (81.10)	1813 (69.70)	<0.001
	Less than two days during the last seven days (*n*, %)	604 (18.90)	789 (30.30)	
Smoking	No (*n*, %)	2626 (82.20)	1840 (70.70)	<0.001
	Yes (*n*, %)	568 (17.80)	762 (29.30)	
Drinking	No alcohol consumption in the last 30 days (*n*, %)	2748 (86.00)	2028 (77.90)	<0.001
	Consumed alcohol in the last 30 days (*n*, %)	446 (14.00)	574 (22.10)	
Exercise	No (*n*, %)	2365 (74.00)	2382 (91.50)	<0.001
	Yes (*n*, %)	829 (26.00)	220 (8.50)	
Household size	Total number of people living in the household (metric variable) (mean ± SD)	2.72 ± 1.29	2.71 ± 1.35	0.914
Social support	In the last 12 months, household did not receive any financial or in-kind support (*n*, %)	2952 (92.40)	1977 (76.70)	<0.001
	In the last 12 months, household received some financial or in-kind support (*n*, %)	242 (7.60)	605 (23.30)	
Regular income	Regular source of income (*n*, %)	3109 (97.30)	1358 (52.20)	<0.001
	Regular but seasonal source of income (*n*, %)	37 (1.20)	825 (31.70)	
	No regular source of income (*n*, %)	48 (1.50)	419 (16.10)	
Debt or loans	Household or any members of the household have current debt or outstanding loans (*n*, %)	178 (5.60)	469 (18.0)	<0.001
	Household or any members of the household have no current debt or outstanding loans (*n*, %)	3016 (94.40)	2133 (82.00)	
Household’s financial situation	Very good (*n*, %)	59 (1.80)	18 (0.70)	<0.001
	Good (*n*, %)	599 (18.80)	227 (8.70)	
	Moderate (*n*, %)	1983 (62.10)	1588 (61.00)	
	Bad (*n*, %)	478 (15.00)	681 (26.20)	
	Very bad (*n*, %)	75 (2.30)	88 (3.40)	
Healthcare expenditure	As a percentage (%) of annual household income (mean ± SD)	0.91 ± 0.29	0.45 ± 0.40	0.048

**Table 2:** Quantile regression results for self-assessed health and quality of life (QOL) using urban data (*n* = 3194).

Variables	Self-Assessed Health						QOL					
	q10		q50		q90		q10		q50		q90	
	Coef.	*p*	Coef.	*p*	Coef.	*p*	Coef.	*p*	Coef.	*p*	Coef.	*p*
Two-week access (ref. = No)												
Yes	1.37	0.019	1.82	0.005	0.08	0.760	−0.39	0.522	0.41	0.457	0.75	0.089
One-year access (ref. = No)												
Yes	−0.95	0.274	−0.44	0.332	0.09	0.739	−0.25	0.785	−0.58	0.197	−1.11	0.052
Access in need (ref. = No)												
Yes	4.97	0.020	1.98	0.001	0.23	0.544	5.11	<0.001	5.12	<0.001	1.89	0.046
Sex (ref. = Male)												
Female	0.17	0.800	−0.61	0.071	0.02	0.892	1.71	0.214	0.21	0.678	0.60	0.183
Age	−0.36	<0.001	−0.24	<0.001	−0.11	<0.001	−0.03	0.470	0.01	0.821	−0.01	0.914
Marital status (ref. = Never married)												
Currently married	6.40	0.008	5.02	0.032	4.97	0.044	6.54	0.112	3.82	0.235	4.09	0.391
Cohabiting	3.37	0.557	3.84	0.169	4.36	0.189	−2.96	0.728	−2.79	0.686	−0.83	0.888
Separated/ divorced	2.07	0.463	2.32	0.339	2.29	0.365	3.50	0.477	0.98	0.803	2.95	0.580
Widowed	4.03	0.119	3.43	0.146	4.40	0.067	6.39	0.110	2.73	0.412	3.05	0.533
Ethnicity (ref. = Han)												
Others	−5.49	0.095	−2.84	0.003	−0.09	0.858	4.48	0.148	0.322	0.792	−2.11	0.055
Education	0.41	<0.001	0.23	<0.001	0.12	<0.001	0.51	<0.001	0.260	<0.001	0.217	<0.001
Living arrangement (ref. = Moved from elsewhere)												
The same place	2.91	<0.001	1.16	<0.001	0.24	0.256	2.29	0.026	1.34	0.004	0.99	0.046
Work (ref. = Yes)												
No	−5.66	0.002	−3.00	<0.001	−1.97	0.010	−3.51	0.049	−3.14	0.019	−0.53	0.643
Smoking (ref. = No)												
Yes	−0.38	0.691	−0.19	0.699	−0.07	0.769	−0.34	0.647	−1.22	0.039	0.48	0.446
Drinking (ref. = No)												
Yes	2.23	0.012	1.54	<0.001	0.34	0.269	3.31	<0.001	1.04	0..064	1.12	0.124
Exercise (ref. = No)												
Yes	3.46	<0.001	1.67	<0.001	0.58	0.020	2.47	0.001	1.62	<0.001	0.39	0.307
Household size	−0.13	0.516	−0.37	0.010	−0.25	0.012	−0.16	0.460	−0.54	0.001	−0.10	0.402
Social support (ref = No)												
Yes	−1.51	0.424	−0.93	0.150	0.16	0.638	−1.75	0.235	−1.82	0.066	−0.31	0.751
Regular income (ref. = Regular)												
Regular but seasonal	−0.75	0.860	0.33	0.836	−0.99	0.355	5.51	0.044	2.87	0.177	2.89	0.045
No	−4.00	0.364	−0.50	0.803	−2.83	<0.001	−2.39	0.459	−5.73	0.159	−0.52	0.892
Debt or loans (ref. = Yes)												
No	3.00	0.086	0.83	0.349	1.05	0.138	1.64	0.412	0.24	0.726	0.09	0.935
Financial situation (ref. = Very good)												
Good	−1.18	0.741	−2.10	0.003	0.69	0.402	−9.29	<0.001	−3.14	<0.001	−10.91	<0.001
Moderate	−2.76	0.411	−2.38	0.001	0.14	0.865	−13.85	<0.001	−6.05	<0.001	−12.87	<0.001
Bad	−5.80	0.093	−4.49	<0.001	−0.98	0.262	−23.26	<0.001	−14.67	<0.001	−15.51	<0.001
Very bad	−8.10	0.060	−7.55	<0.001	−3.25	0.025	−26.72	<0.001	−17.06	<0.001	−14.86	<0.001
Healthcare expenditure	−0.02	0.988	−0.01	0.964	−0.01	0.958	−0.01	0.788	−0.01	0.988	−0.01	0.787
Pseudo R^2^	0.15		0.13		0.07		0.17		0.12		0.05	

Note: models were established based on 2000 bootstrap samples.

**Table 3:** Quantile regression results for self-assessed health and QOL using rural data (*n* = 2602).

Variables	Self-Assessed Health						QOL					
	q10		q50		q90		q10		q50		q90	
	Coef.	*p*	Coef.	*p*	Coef.	*p*	Coef.	*p*	Coef.	*p*	Coef.	*p*
Two-week access (ref. = No)												
Yes	−1.63	0.087	0.67	0.157	0.10	0.796	−1.26	0.206	0.20	0.806	2.10	0.042
One-year access (ref. = No)												
Yes	1.74	0.097	−0.08	0.878	−0.19	0.635	1.44	0.032	0.05	0.927	−1.45	0.360
Access in need (ref. = No)												
Yes	3.83	0.006	3.43	<0.001	1.73	0.004	1.27	0.493	1.23	0.379	0.87	0.555
Sex (ref. = Male)												
Female	−0.61	0.645	−0.57	0.344	−1.05	0.041	−1.25	0.188	1.15	0.186	0.88	0.392
Age	−0.52	<0.001	−0.26	<0.001	−0.13	<0.001	−0.21	<0.001	−0.13	0.002	−0.04	0.355
Marital status (ref. = Never married)												
Currently married	18.45	0.013	8.59	0.016	−3.00	0.543	3.61	0.564	6.38	0.023	−0.65	0.909
Cohabiting	5.36	0.695	12.40	0.002	−3.14	0.489	4.15	0.710	13.92	0.026	12.49	0.133
Separated/divorced	22.98	0.007	7.96	0.032	−2.28	0.665	3.84	0.574	4.47	0.277	−3.42	0.563
Widowed	18.03	0.020	8.24	0.025	−3.25	0.513	3.71	0.562	5.71	0.058	−1.32	0.821
Ethnicity (ref. = Han)												
Others	1.73	0.678	1.00	0.351	1.74	0.400	1.50	0.698	2.43	0.436	−0.54	0.891
Education	0.21	0.061	0.13	0.037	0.07	0.114	0.16	0.123	0.29	0.007	0.39	0.006
Living arrangement (ref. = Moved from elsewhere)												
The same place	1.50	0.056	0.94	0.101	0.16	0.609	−0.57	0.440	−0.06	0.937	0.377	0.643
Work (ref. = Yes)												
No	−5.17	0.004	−3.57	<0.001	−0.75	0.096	−3.51	0.034	−5.25	<0.001	−0.92	0.431
Smoking (ref. = No)												
Yes	0.44	0.755	0.53	0.411	−0.63	0.245	−1.83	0.056	0.21	0.819	−0.26	0.779
Drinking (ref = No)												
Yes	3.48	<0.001	1.28	0.008	0.22	0.615	3.14	0.001	2.48	0.001	1.45	0.097
Exercise (ref. = No)												
Yes	3.95	0.003	0.85	0.285	0.67	0.319	4.41	0.002	3.34	<0.001	1.61	0.244
Household size	0.59	0.134	0.61	<0.001	0.32	0.011	0.19	0.413	0.42	0.067	0.64	0.019
Social support (ref. = No)												
Yes	−1.53	0.134	−2.54	<0.001	−1.36	0.004	0.60	0.544	0.64	0.347	−0.37	0.665
Regular income (ref. = Regular)												
Regular but seasonal	−0.54	0.660	−0.40	0.376	−0.48	0.205	−2.49	0.001	−1.21	0.084	−2.15	0.005
No	−4.19	0.041	−2.41	<0.001	−1.09	0.010	−4.70	<0.001	−2.41	0.018	−2.69	0.012
Debt or loans (ref. = Yes)												
No	5.30	<0.001	1.82	<0.001	0.73	0.167	3.56	0.001	2.90	0.015	1.83	0.048
Financial situation (ref. = Very good)												
Good	0.60	0.901	−0.98	0.469	−0.60	0.691	−4.91	0.488	0.65	0.855	−9.26	0.170
Moderate	−2.01	0.698	−3.28	0.008	−1.65	0.242	−9.99	0.147	−4.00	0.255	−14.37	0.028
Bad	−4.70	0.375	−4.75	<0.001	−3.10	0.028	−13.97	0.044	−10.06	0.005	−16.62	0.010
Very bad	−9.90	0.059	−9.52	<0.001	−3.33	0.019	−21.66	0.004	−15.86	<0.001	−21.70	0.002
Healthcare expenditure	0.05	0.832	−0.03	0.408	−0.04	0.151	−0.02	0.813	−0.08	0.285	−0.17	<0.001
Pseudo R^2^	0.19		0.12		0.07		0.13		0.10		0.06	

Note: models were established based on 2000 bootstrap samples.
